# An Asymptomatic Intracranial Foreign Body in a Child as a Result of Unintentional Firearm Injury

**DOI:** 10.7759/cureus.16757

**Published:** 2021-07-30

**Authors:** Mohammad J Faisal, Muhammad Y Wajid, Mahnoor Shahid, Muhammad H Anwar, Haaris Aamer

**Affiliations:** 1 Surgery, Fauji Foundation Hospital (FFH), Rawalpindi, PAK; 2 Surgery, Foundation University Medical College, Rawalpindi, PAK

**Keywords:** intracranial foreign body, asymptomatic presentation, bullet, trauma, head injury, x-ray skull, ct brain

## Abstract

Aerial firing is often used as a form of riot control, but also in certain parts of the world as a celebratory act, often resulting in unintentional injury and/or death. It is uncommon for a patient to walk into an emergency room, seemingly comfortable, only to discover an intracranial foreign body. We report a case of a child who was struck by a stray bullet that pierced his skull through a small entry wound and lodged itself in the falx cerebri. The patient remained asymptomatic and no intervention was required. We wish to highlight the importance of imaging techniques in patients with small wounds who are otherwise asymptomatic as well as point out the salient features regarding stray bullet injuries.

## Introduction

Stray bullet injuries may arise from a vast multitude of scenarios, one of which includes aerial firing. They are categorised as a form of unintentional firearm injury, the incidence of which has increased over the past few decades [1]. While some patients may present with weakness or signs of raised intracranial pressure, we came across a peculiar case, in which the victim presented with no signs of neurological impairment. It may often be difficult to appreciate the presence of a foreign body in these cases, especially as many patients are unaware they have sustained a gunshot injury especially if the inlet injury is small [2,3]. Therefore, we cannot undermine the significance of radiological imaging. In particular, the use of CT imaging has become increasingly crucial in such cases [4]. Moreover, it is important to understand that the potential early and late complications of an intracranial foreign body are numerous, thus portraying the need for regular follow-up [4,5]. With this case report, we wish to highlight the importance of imaging techniques in asymptomatic patients with small wounds when there is a clinical suspicion of a foreign body as well as point out the salient features regarding stray bullet injuries.

## Case presentation

An 11-year-old boy presented to the emergency department of a tertiary care hospital in Pakistan, claiming an unknown object had struck his forehead. This resulted in extensive bleeding that was controlled by the application of a pressure dressing, which was performed by paramedics in the ambulance. The wound edges were regular, slit-like, measuring 5mm. Both the family and the patient were not sure of what had struck the child.

**Figure 1:**
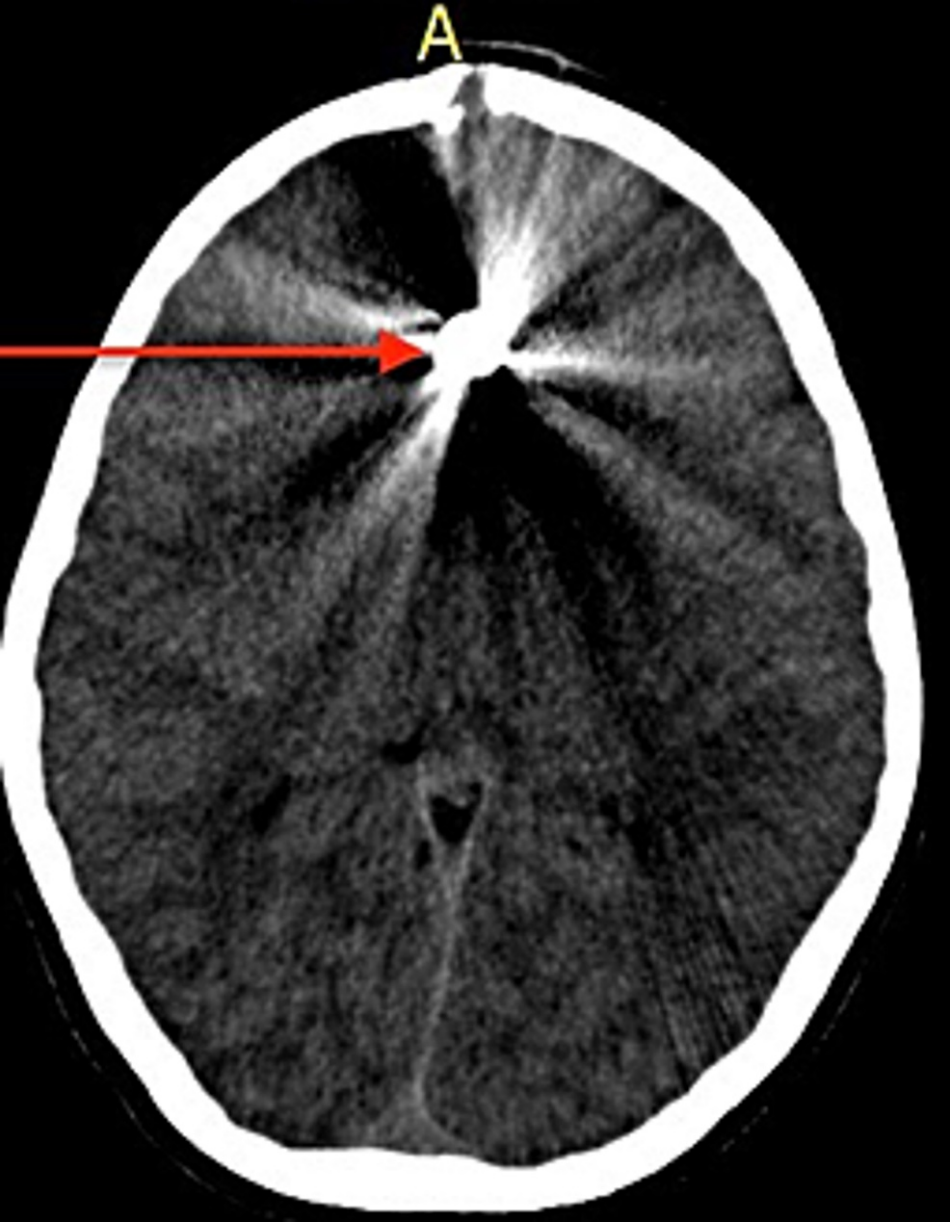
Computed tomography scan of the head showing an axial view in which a metallic foreign body is seen with a beam hardening artefact

**Figure 2:**
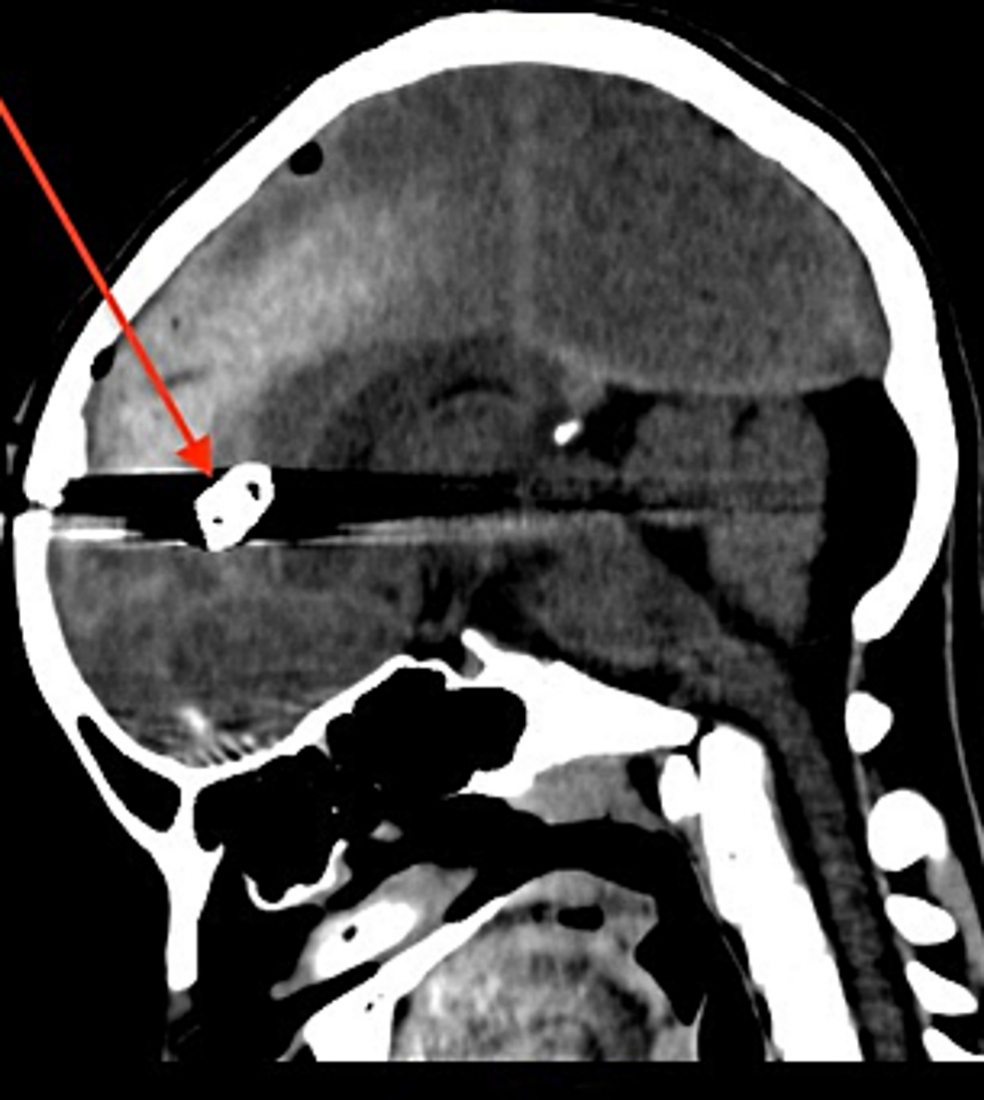
Computed tomography scan of the head showing the metallic foreign body with surrounding pneumocephalus

On physical examination, he was vitally stable, conscious and alert. A full neurological examination was performed, which revealed no loss of sensory, motor, or cerebellar function, along with normal reflexes and was thus deemed neurologically intact. In addition, the patient had no history of loss of consciousness, altered sensorium, weakness, vomiting, ENT bleeding, confusion or amnesia. Furthermore, a fundoscopy revealed a normal fundus. An x-ray skull was ordered, which surprisingly, revealed a bullet inside the skull. Given the size of the wound, this came as a surprise. A non-contrast CT scan of the brain was then performed to confirm the location of the bullet and to assess for any intracranial bleeding, air or other abnormality resulting from the injury. The soft tissue and bone windows of the CT demonstrated a radiopaque foreign body in the anterior interhemispheric cisterns along the falx cerebri, with an extensive beam hardening artefact and a trace amount of pneumocephalus along the falx cerebri. There was no significant haemorrhage or soft tissue oedema. The location of the entry wound was confirmed to be in the frontal bone, with minimally displaced ossific fragments (Figures 1, 2). A CT angiography would have been a more accurate tool to observe vascular abnormalities and/or intracranial haemorrhage; however, due to limited resources, it could not be performed. 

The patient and his family were counselled regarding the situation, the findings and potential complications such as haemorrhage, infection and dislodging of the bullet. They had explained how they sat outside, listening to the music from a nearby wedding event and how it is not uncommon for people in his area to fire rounds of bullets into the sky as a form of celebration.

The wound was washed and redressed, after which he was admitted to the Neurosurgery ward. A set of baseline laboratory investigations were all sent, the only abnormal value being a mildly elevated alkaline phosphatase level, which can be attributed to the injury sustained to the cranium. His treatment included amoxicillin/clavulanic acid and sodium valproate, as protection against infection and convulsions respectively. He was strictly observed and examined repeatedly overnight and the days to follow. Eventually, the patient was discharged and asked to look out for any signs of weakness, fever, discharge from the wound, vomiting, altered consciousness, personality changes and/or visual disturbances. Emphasis was made on weekly follow-up. The decision to not intervene was made based upon the consistently normal neurological examination findings.

The findings emphasise the importance of imaging in traumatic head injuries (even with small wounds), as the possibility of intracranial foreign bodies in an asymptomatic patient cannot be ruled out in any other way. Such imaging could prove useful in injuries sustained far from the initial site of offense (e.g., secondary blast injuries or shrapnel injuries).

## Discussion

It has been noted, that the incidence of unintentional firearm injuries has increased over the last few decades [1,2]. This can often lead to grave circumstances and grim outcomes. Aerial firing is often used as a means of riot control, or as a celebratory act in some parts of the world; meanwhile, other causes of unintentional firearm injuries include poor weapon handling, accidents during cleaning of the firearm, lack of weapon safety devices, attempted suicides and firing by playing children [1]. A similar spectrum of injury can be expected from secondary blast and shrapnel injuries.

Stray bullet injuries have been noted in many studies. It is the second most common cause of injury-related deaths in adolescents, the first being motor vehicle accidents [6]. One study, in particular, mentioned the site of injury most commonly associated with death to be that of the central nervous system, with regards to stray bullet injuries [1].

The study of ballistics is extensive and complicated; however, it is worth noting the importance of the ‘entry wound’. Generally, the wound at the entry points, are small with more regular margins [3]. The smaller wounds may make it difficult to appreciate the possibility of an unintentional aerial projectile-related injury. Furthermore, victims of these injuries may be unaware that the injuries they have sustained are due to firearms [2]. Therefore, the importance of appropriate imaging to identify the presence of a foreign body is emphasised.

Urgent imaging would be a certain step in the diagnostic protocol in a patient who is haemodynamically stable. The appearance of a metallic bullet on an x-ray would be radiopaque, thus making x-ray imaging a useful screening tool in the initial detection of the foreign body. In addition, these plain radiographs provide details of fractures near the points of entry and exit. However, non-contrast-enhanced CT is now part of the emergency imaging for craniocerebral injury, even if the clinical examination lacks supportive findings for intracranial penetration [4]. With the advances in medical technology in centres that are well equipped, multidetector-row CT (MDCT) angiography has proven to be ground-breaking in the initial assessment of such cases, providing more information about soft tissue structures and vascular injury [6]. The use of MRI has been proposed; however, given the ferromagnetic nature of most bullets and the uncertainty of the magnetic composition of bullets, it is not advised as it may potentiate migration of the bullet [4].

Aside from the early manifestations of sustaining a bullet wound, there are several complications that can take place in a delayed manner, as a result of an intracranial foreign body, which highlights the need for regular follow-up in such cases, regardless of the symptomatic status of the patient. There have been many reports of spontaneous bullet migration, intracranially, the earliest report of this being within 36 hours of the initial trauma [5]. There has also been mention of intracranial abscess formation, ventriculitis, obstructive hydrocephalus, cerebrospinal fluid (CSF) fistulas, cerebral herniation epilepsy, bullet embolisms and even lead toxicity [4,7-9].

There is limited literature on the management protocol for asymptomatic cases. In the event of an intracranial foreign body, the decision to operate has always been a controversial topic but is based on many factors including the site of the foreign body, symptomatology of the patient, and whether it is accessible and can be retrieved safely [7]. Glasgow Coma Scale (GCS) on admission showed the presence of an operable or expanding hematoma, and potential post-operative life-changing complications are also taken into account [8,10].

## Conclusions

Stray bullet injuries may arise from a vast multitude of scenarios. In the event of unintentional firearm injury, one cannot rule out the presence of an intracranial foreign body based on clinical examination alone. In fact, many patients themselves are unaware of the fact that they have been struck by a bullet at all. We can expect similar features in those with shrapnel or secondary blast injuries. The key to diagnosing these cases lies in the use of x-rays and CT imaging, in particular MDCT angiography. X-rays confirm the presence and provide information about the entrance wound and skeletal injury, and CT and MDCT scans inform us about the exact localisation, track and damage caused. While there are many potential complications of an asymptomatic intracranial metallic foreign body, the decision to intervene depends on the surgeon’s clinical judgement of the situation.
